# Multicomponent Physical Exercise in Older Adults after Hospitalization: A Randomized Controlled Trial Comparing Short- vs. Long-Term Group-Based Interventions

**DOI:** 10.3390/ijerph17020666

**Published:** 2020-01-20

**Authors:** Iñaki Echeverria, Maria Amasene, Miriam Urquiza, Idoia Labayen, Pilar Anaut, Ana Rodriguez-Larrad, Jon Irazusta, Ariadna Besga

**Affiliations:** 1 Department of Physiology, University of the Basque Country, UPV/EHU, 48940 Leioa, Spain; miriam.urquiza@ehu.eus (M.U.); ana.rodriguez@ehu.eus (A.R.-L.); jon.irazusta@ehu.eus (J.I.); 2 Department of RMSB, UMR 5536, Université Bordeaux/CNRS, 33000 Bordeaux, France; 3 Department of Pharmacy and Food Science, University of the Basque Country UPV/EHU, 01006 Vitoria-Gasteiz, Spain; maria.amasene@ehu.eus; 4 Institute for Innovation & Sustainable Development in Food Chain (IS-FOOD), Public University of Navarra, 31006 Pamplona, Spain; idoia.labayen@unavarra.es; 5 Department of Medicine, Araba University Hospital, Bioaraba Research Institute, OSI Araba, CIBERSAM, University of the Basque Country (UPV/EHU), 01004 Vitoria-Gasteiz, Spain; mariapilar.anautmayo@osakidetza.eus (P.A.); ariadna.besgabasterra@osakidetza.eus (A.B.)

**Keywords:** post-hospitalization, older adults, multicomponent exercise program, physical function, nutrition, quality of life

## Abstract

Multicomponent physical exercise is effective in curbing the effect of hospitalization in older adults. However, it is not well established which characteristics of the exercise interventions would optimize intervention sustainability and efficacy. This study compared the effects of two group-based multicomponent exercise interventions of different lengths in older adults after hospitalization. Fifty-five participants were randomly assigned to a short-term group-based branch (SGB, *n* = 27) or to a long-term group-based branch (LGB, *n* = 28). The SGB participated in a six-week multicomponent group-based exercise-training program followed by 18 weeks of home-based exercise. The LGB completed 12 weeks of each phase. Physical function, physical activity, quality of life, anthropometrics, and nutritional status were assessed at baseline, after 12 weeks, and after 24 weeks of intervention. Both groups improved physical function and nutritional status and increased physical activity after 12 weeks of intervention (paired student’s *t*-test, *p* < 0.01), and maintained the positive effects during the following 12 weeks. No group-by-time interaction was observed in any of the studied variables using mixed-model ANOVA. Based on these findings, we determined that 6 weeks of a group-based exercise intervention caused similar functional and nutritional benefits to a longer group-based intervention of 12 weeks when both are continued at home until 24 weeks.

## 1. Introduction

By 2050, the percentage of the world’s population that is over age 60 will reach an estimated 22% due to an increase in life expectancy [1]. The process of aging encompasses changes in body composition, with reductions in muscle and bone mass and increases in fat mass, leading to functional performance decline [2]. This progressive loss of functional performance is one of the most important health-related problems linked to aging. Low physical function is related to a high incidence of malnutrition, falls, high morbidity, mortality, and disability, along with reductions in quality of life [3,4]. Thus, the loss of physical function represents a challenge for older adults and carries an associated increase in health-related resource demand and economic burden [5].

The addition of stress to the aging process, due to illness or a hospitalization, usually accelerates deconditioning. Hospitalization in older patients is associated with decreased muscle strength and muscle mass loss and worsens nutritional status and quality of life [6]. The strong association between stress and deterioration can increase the risk of negative outcomes such as dependence or mortality [7,8,9], and this particularly affects the oldest patients [10]. This decline in capacities may continue months after discharge [10,11], even more than one year following hospital admission [12], and without appropriate intervention, hospital-associated deconditioning has the potential to chronically precipitate poor outcomes [13,14].

Multicomponent physical exercise programs including strength, endurance, and balance training are effective in attenuating the adverse effects associated with aging [15,16,17]. When performed during or after acute hospitalization, these interventions increase muscle strength and functional capacity and are effective in improving physical function and cognitive status [18,19,20,21]. However, exercise interventions are often difficult to implement during hospitalization due to disease severity and the complexity of medical care. Most post-hospitalization physical exercise programs are utilized with patients undergoing cardiac and lung pathologies or after hip or knee repair [22,23,24]. However, few studies assess post-hospitalization exercise programs in older adults with conditions for which exercise is not considered part of the treatment [19,25]. In addition, the social and economic cost of physical exercise interventions is a cause of concern. Therefore, effective and sustainable interventions to accelerate the recovery of physical function after hospital discharge are warranted [26].

Most physical exercise interventions could be categorized as group-based or home-based. In a clinical setting, group-based exercise is monitored by qualified staff who ensure attention to exercise technique and proper exercise performance, allowing for increases in and optimization of the loads and difficulty of the exercises and facilitate follow-up [27,28]. However, experienced trainers and transport to facilities are necessary, making these programs more difficult to maintain long-term. In contrast, home-based exercise programs have some benefits over group-based exercise approaches, including the tailoring of exercise to lifestyle preferences, participant autonomy, flexibility in timing, low cost to the individual, and no need for travel. Nevertheless, there are some limitations to home-based programs, including the need for strong self-discipline to adhere to the program and lack of a social element of the program (the latter is considered a positive element of group exercise) [29]. Typically, the higher the intensity of supervision, the greater the improvements [30,31], but there is no consensus on what programs provide the greatest improvements in older adults. In some programs, after initial supervised sessions to facilitate performance and adherence, there is a period of home-based sessions. These programs can take advantage of both types of interventions. In fact, more positive effects in the long-term were found when there was follow-up at home or in a community after finishing a group-based intervention [14,32,33]. In this case, the length of the group-based component could be a limiting factor due to its economic and social cost. Hence, high-quality randomized controlled trials have been proposed to evaluate graded approaches, implementing different stages of exercise supervision [34].

Therefore, we aimed to compare the benefits of two multicomponent physical exercise interventions with different group- and home-based period lengths on physical function, physical activity, quality of life, anthropometry, and nutritional status among older adults after hospitalization.

## 2. Materials and Methods

### 2.1. Study Design

This was a 24-week single-blinded randomized controlled intervention study. Participants were randomly assigned to either a short-term group-based branch (SGB) or a long-term group-based branch (LGB). The SGB consisted of six weeks of group sessions in the hospital and 18 weeks of individual sessions at home. The LGB undertook a 12-week group exercise session in the hospital with an additional 12 weeks completed at home. The study was approved by the Committee on Ethics in Research at the University Hospital of Araba (CEIC-HUA Code Expte. 2017-021). Written informed consent was obtained from all study participants before enrollment in the study. The protocol was registered under the Australian and New Zealand Clinical Trials Registry (ANZCTR) with the identifier ACTRN12619000093189.

Participants were recruited from the Internal Medicine and Neurology services of the University Hospital of Araba (HUA). Eligible participants were aged ≥70 years, scored ≥20 on the Mini Mental State Examination (MMSE) [35], and were able to walk (with or without assistive devices) independently for at least 4 m. All participants provided informed consent. Exclusion criteria were a diagnosis of chronic kidney disease, severe dementia, autoimmune neuromuscular disease, acute myocardial infarction or a bone fracture in the last three months, or refusal to sign the informed consent form.

**Figure 1:**
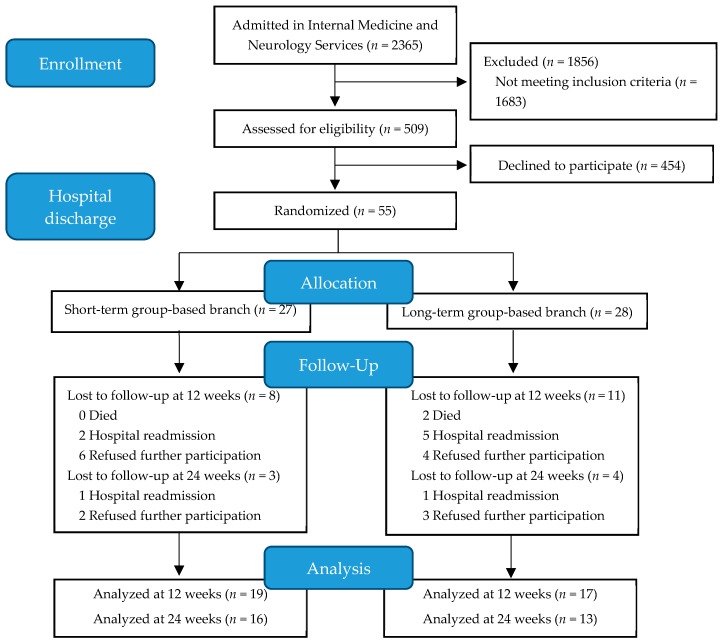
Flow diagram of participant selection.

In total, 2365 patients were admitted during the recruitment period. Once screened, 509 patients were eligible to initiate the physical exercise program at discharge. After signing the informed consent document, participants were evaluated in their hospital room and prepped to start a physical exercise program at the same hospital at discharge. Among these evaluated patients, 55 accepted participation in the physical exercise program at discharge. Participants were randomly assigned (in a 1:1 ratio) using sealed opaque envelopes to either the SGB or LGB by coin-toss sequence generation. All volunteers received detailed study information through the research team; objectives, measurement variables, and other details about the interventions were explained orally and in writing to both potential participants and their families. Finally, 27 patients were assigned to the SGB and 28 to the LGB. A flow diagram describing the recruitment of participants is shown in Figure 1.

### 2.2. Group-Based and Home-Based Interventions

The group-based intervention consisted of multicomponent physical exercise sessions designed to improve strength, power, balance, and walking conducted by experienced physical trainers. The program’s technical content was based on the authors’ previous experience in a population with a high prevalence of frail older adults [36]. The intervention was adapted to meet the exercise and physical activity guidelines for older adults established by the American College of Sports Medicine (ACSM) and the American Heart Association (AHA) [37,38]. The program was individualized based on each participant’s physical function, and progression was set up accordingly.

The group-based program was performed in a room equipped ad hoc at the hospital and consisted of 60-min group sessions conducted twice a week with exercises intended to improve strength, power, and balance. An interval of at least 48 h between training sessions was respected. All sessions began with a brief warm-up of 5 min (range-of-motion exercises for the neck, wrists, shoulders, hip, knees, and ankles). Strength training (35 min) comprised upper- and lower-limb exercises (arm curl, leg flexion, hip extension, leg abduction, standing on tip-toes and heels, and chair stand) performed with external weights and tailored to each participant´s physical function via the Brzycki equation [39] for the estimation of one-repetition maximum (1-RM). In the first three weeks, exercise was performed with light loads (40–50% 1-RM) to ensure an appropriate introduction and familiarization with resistance training exercises, and thereafter, loads were increased to 60–70% of each participant´s 1-RM, to obtain additional benefits if they were well tolerated.

Balance training (for 15 min) included exercises of progressing difficulty, starting with decreasing hand support (with two hands at first, then with one hand, and finally, none, if possible) along with decreasing the base of support (both feet together, semi-tandem, and tandem position, while increasing the complexity of movements). The progression of each balance exercise was adapted and individualized weekly and exercises varied. Typical exercises were weight transfer from one leg to another, walking with small obstacles, proprioceptive exercises, and stepping practice. Sessions finished with five minutes of cooling down by stretching, breathing, and relaxing. Training attendance was recorded for every session. A session was considered complete when 80% or more of the programmed exercises were performed.

The home-based intervention was influenced by the Otago Exercise Program and the European Vivifrail project [40,41]. The schedule for each week and patient consisted of five days of multicomponent exercises and seven days of walking recommendations. Exercises were learned during the group-based period. Walking retraining was also implemented through individualized recommendations, with the goal of performing outdoor walking sessions without assistance. Walking recommendations started with 15 min per day at the beginning of the intervention with the goal of completing 45–60 min/day by the end of the program. Walking intensities were based on each participant’s 6-min walk test performance. Every two weeks, the staff supported the participants, addressed any concerns, and registered adherence via phone call. In this case, there were five sessions carried out per week rather than two in the group-based period. Compliance with the multicomponent exercise was considered successful when each subject performed 50% or more of the session.

### 2.3. Measurements

**Figure 2:**
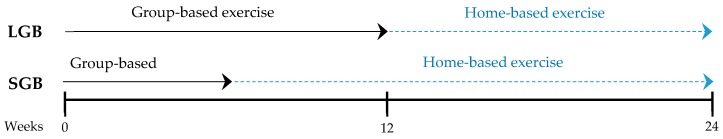
Exercise program duration and measurements weeks.

Measurements were recorded in both groups before the intervention began (week 0), at the end of the long group-based phase (week 12), and after the full intervention period (week 24; Figure 2). All measurements were evaluated at the same location, and all outcomes were collected by the same trained researchers.

#### 2.3.1. Socio-Demographic and Clinical Data

The following data were retrieved from the Basque Public Health Service’s database: patient demographic data (sex and age), comorbidity [assessed through the Charlson Comorbidity Index (CCI)], and basic and instrumental activities of daily living (Barthel and Lawton Indexes). Socio-demographic data including patients educational level, whether or not they live alone, use of assistive devices for walking, and home accessibility (defined as not having home entrance-related environmental barriers, i.e., not having elevator/lift or ramp) were also collected.

#### 2.3.2. Physical Function, Physical Activity, and Quality of Life

The primary outcome was functional capacity, measured by the Short Physical Performance Battery (SPPB) [42]. Physical function assessment included a handgrip strength test (Jamar dynamometer) [43], balance per the Berg scale [44], normal and fast 8-m walking speed [45], and four tests from the Senior Fitness Test: the 30-s chair-stand test (CST), the arm-curl test (ACT), 8-ft timed up-and-go test (8-ft TUGT), and the 6-min walk test (6mWT) [46]. These tests were used to assess functional capacity, lower- and upper-limb strength, gait speed, static and dynamic balance, and aerobic endurance. 

Time spent at different intensities of physical activity and the number of steps per day during free living of everyday life were recorded with an accelerometer (Actigraph GT3X model; Actigraph LLC, Pensacola, FL, USA) that participants wore on the hip with a belt for a seven-day period. Active-period intensities were classified as light, moderate, or vigorous following the criteria developed by Freedson et al., and the number of minutes performed at each intensity was measured [47]. Participants did not receive specific instructions to walk during the assessments.

Quality of life (QoL) was assessed using the Spanish version of the EuroQol–5 Dimension (EQ-5D-5L) questionnaire. EQ-5D-5L combines the assessment of the visual analog scale of the self-rated health score (EQ VAS Score) and the health index (EQ-5D-5L Index Value) [48].

#### 2.3.3. Anthropometry and Nutritional Status

Anthropometry measurements were obtained before functional assessments. An experienced nutritionist who was internationally certified in anthropometric testing (ISAK level 1) obtained all anthropometric measurements following the protocol recommended by the International Society for the Advancement of Kinanthropometry [49].

Height was measured with a Seca213 stadiometer to the nearest 0.1 cm, and body mass was measured with an OmronHN288 digital scale to the nearest 0.1 kg. Calf, arm, waist, and hip circumference was measured with non-elastic anthropometric tape (CESCORF) to the nearest 0.1 cm. Body mass index (BMI) was calculated based on height and mass, and the waist-to-hip ratio was based on waist and hip circumferences.

Nutritional status was evaluated qualitatively using the Mini Nutritional Assessment test (MNA) [50], which was completed by the participant and/or the participant’s relative or caregiver. This questionnaire contains 18 items divided into four categories: anthropometric assessment, general assessment, short dietary assessment, and subjective assessment. Each answer has a numerical value contributing to the final score. A maximum of 30 points can be obtained. Scores ranging from 24 to 30 reflect normal nutritional status, scores from 17 to 23.5 reflect a risk of malnutrition, and a score under 17 reflects malnutrition [50].

### 2.4. Sample Size and Statistical Analyses

Sample size was calculated to detect minimal significant effects on SPPB accepting an alpha risk of 0.05 and a beta risk of 0.20 in a bilateral contrast. A total of 63 individuals were required to detect a difference equal to or greater than 1 unit in the SPPB (SD = 2.00). The sample size was increased, with an additional 20% (loss during follow-up) and 5% (mortality). The resultant sample size required was 84 individuals or 42 individuals per group.

Qualitative data are presented as percentages. Chi-squared test was used to compare two groups at baseline. Quantitative data are presented as mean ± SD. Normal distribution of the data was determined by the Shapiro–Wilk test. When not normally distributed, the data were square-root transformed. Comparisons of quantitative data between two groups at baseline were performed using an unpaired *t*-test and chi-squared.

Group-by-time differences were assessed using mixed design analysis of variance (ANOVA; two time points × two groups) with two different levels (weeks 0–12 and weeks 12–24). Eta squared (η^2^) was calculated to estimate the effect size. Values for η^2^ of ≤0.02, ≤0.13, ≥013, and ≥0.26 were considered small, medium, medium-large and large, respectively [51]. Comparisons of the changes within each group were performed with paired Student’s t-tests. Thresholds of 0.1, 0.3, 0.5, 0.7, and 0.9 were used for small, moderate, large, very large, and extremely large effects, respectively, as suggested by Cohen [52]. Relationships between categorical variables (contingency tables) were determined by a chi-square test (χ^2^).

The significance level for all tests was *p* < 0.05. Statistical analysis was performed using the IBM SPSS Statistics 24 statistical software package (SPSS, Inc., Chicago, IL, USA).

## 3. Results

### 3.1. Study Participants

From September 2017 to July 2018, a total of 509 (21.5%) patients admitted to the Internal Medicine and Neurology services of the HUA were screened for eligibility. Among the evaluated patients, 55 started the program (Figure 1). According to hospital admission records, 41.8% admissions were infection-related, 18.2% were due to stroke without motor impairment, 16.4% were decompensated heart failure admissions, and 5.5% were due to falls. The remaining 18% of the admissions were due to other conditions (neurological, delirium, anemia, etc.). Of the 55 patients (26 females, 47.3%) that were initially enrolled, 19 (SGB = 8 and LGB = 11) were lost to follow-up before the 12-week time point. The total number of subjects lost to follow-up at the 24-week time point was 26 (SGB = 11 and LGB = 15, Figure 1). The descriptive characteristics of the study participants are explained in Table 1. No significant differences between the two groups existed at baseline. The program was ceased before reaching the estimated sample size because the difficulties in the recruitment. In addition, at that moment, the primary outcome measure was far from the expected difference between the two assessed interventions.

### 3.2. Adherence and Compliance

Mean attendance rates for the group-based exercise sessions were 1.81 days/week (90.4% of the sessions) in the SGB and 1.74 days/week (87% of the sessions) in the LGB. Compliance with the walking recommendation was 6.92 days/week (97.6% of the days) in the SGB and 5.63 days/week (80.4% of the days) in the LGB. During the home-based period, the SGB performed the exercises 4.1 days/week (82.3% of the sessions) and the LGB performed the exercises 3.4 days/week (67.9% of the sessions) on average. There was no significant difference between groups, except in walking recommendation compliance (*p* = 0.008). No adverse events associated with the prescribed exercises were recorded, and no patients had to interrupt the intervention because of adverse events.

### 3.3. Physical Function, Physical Activity, and Quality of Life Outcomes

Significant improvements were obtained in almost all parameters in both groups from baseline through week 12 (Table 2). A clinically and statistically significant increase was observed in the main outcome of this trial, the SPPB score (SGB *p* < 0.001; Cohen’s d = extremely large, and LGB *p* < 0.001; Cohen’s d = extremely large). Similarly, the strength of the lower and upper limbs (except for the grip strength of both hands), fast and normal gait speed, static and dynamic balance, and aerobic endurance also increased in both branches after 12 weeks of the intervention. These improvements were maintained from week 12 to week 24 (Table 3). We did not find any significant group-by-time interaction effect in either period (0–12 weeks and 12–24 weeks, Table 2 and Table 3).

LGB participants in the 0–12 period increased light physical activity (*p* = 0.004; Cohen’s d = very large) and the number of steps per day (*p* = 0.015; Cohen’s d = large). In contrast, SGB participants increased moderate to vigorous physical activity (MVPA) (*p* = 0.026; Cohen′s d = large) during this period. We did not find any significant group-by-time interaction effect in either period (0–12 weeks and 12–24 weeks, Table 2 and Table 3). 

The LGB showed significant improvement in self-rated health (*p* = 0.027; Cohen’s d = large) and in the health index (*p* = 0.029; Cohen’s d = large) after the first 12 weeks of intervention. There was no significant difference within the SGB. From weeks 12 to 24, when both groups performed home-based physical exercise, there were no significant differences in QoL variables, although there was a trend toward QoL reduction (*p* = 0.201; Cohen’s d = moderate) among the LGB.

### 3.4. Anthropometry and Nutritional Status Outcomes

After the first 12 weeks, both branches improved almost three points on average in the MNA test (SGB 2.92; *p* < 0.001; Cohen’s d = extremely large and LGB 2.85; *p* = 0.001; Cohen’s d = extremely large). The SGB increased the right (*p* = 0.043; Cohen’s d = large) and the left calf (*p* = 0.039; Cohen’s d = moderate) perimeter from baseline to week 12. The MNA ranges significantly changed over time in the SGB (*p* = 0.004; χ^2^ = 11.1) and LGB (*p* = 0.004; χ^2^ = 0.022). At the beginning of the intervention, most participants were in the at-risk and malnutrition ranges. However, by the end of the long group-based period, most participants had a normal nutritional state. Thus, 52.6% of participants in the SGB and 41.2% of participants in the LGB improved their nutritional status from being at risk of malnutrition to having a normal nutritional status. In the LGB, there was a 17.6% reduction in participants with malnourished status, leaving no participants in this range at the end of the intervention. However, we did not find any significant group-by-time interaction. There was no difference from baseline to 12 weeks in either group in the rest of the anthropometric measurements (Table 4).

From weeks 12 to 24, when both groups performed home-based exercise, we did not find any significant difference in the MNA score and point range or anthropometrical parameters within either group or between the two groups. Nevertheless, it was noticeable that both branches maintained the improvements in nutritional status obtained during the first 12 weeks of the intervention (Table 5). 

## 4. Discussion

In the present study, both multicomponent physical exercise interventions implemented at hospital discharge provided significant benefits in physical function and nutritional status, combating the functional decline associated with hospitalization in older adults. The results also suggest that a six-week group-based multicomponent exercise intervention is sufficient to reverse the functional and nutritional decline associated with hospitalization.

Considering that we did not have a control group, it could be that physical improvements observed after hospitalization were simply due to overcoming the acute condition. However, previous studies have demonstrated that in the absence of physical intervention, SPPB score does not improve significantly in the months after hospitalization [18,30,53]. SPPB is a valid instrument for screening the risk of disability [42] and provides important prognostic information to identify older adults at high risk of poor outcomes after hospital discharge [54]. In this study, the SPPB score increased approximately two points during the first 12 weeks of both interventions (only one point is clinically relevant [55]) and these improvements were maintained during the next 12 weeks of home-based intervention. The improvement observed in physical function after both interventions indicates that the assessed programs are effective in reducing the risk of poor outcomes after hospital discharge.

Studies assessing exercise programs to improve recovery after hospital discharge have reported conflicting results regarding their benefits in physical function. The type of intervention (group versus home-based) and the pathologies of the patients could explain this contradiction. Our results agree with previous works showing that group-based multicomponent programs are efficient in ameliorating or reversing the functional decline associated with hospitalization [18]. In contrast, home-based interventions show more modest results [28,29,53]. The level of supervision seems to be relevant in home-based programs to obtain greater improvements in physical function [27,28,29]. Some authors propose that the smaller effects of home-based programs on strength could be due to the difficulty of delivering programs at a sufficient intensity in a home setting [53]. Nevertheless, it has been suggested that the recovery of patients’ physical function and activity of daily living could be further benefited by a prolonged in-home intervention after exercise programs during hospitalization [32,33]. We observed that home-based physical exercise maintained the physical function and activity achieved in both group-based periods. Our results also agree with previous reports showing that home visits and follow-up telephone calls are effective in maintaining adherence and compliance in home-based exercise [28,33].

Notably, the MNA score improved after the first 12 weeks of intervention. At baseline, both groups were at average risk of malnourishment. After 12 weeks, the mean score in the SGB (25 points) achieved good nutritional status and the LGB was close to having good nutritional status (23.9 points). Accordingly, while most of the participants were at risk of malnutrition at the beginning of the intervention, after 12 weeks, those with good nutritional status outnumbered those at risk of malnutrition and only one subject was malnourished according to the MNA. Notably, this nutritional improvement was maintained in the following three months, with the proportion of participants at an optimal state of nutrition reaching 82.8%. To our knowledge, this is the first study to describe the effects of post-hospitalization interventions on MNA. However, studies performed in other populations demonstrate the effectiveness of physical exercise in improving nutritional status [56]. Our results show that nutritional status was within an acceptable range at the end of the intervention in both groups, suggesting that physical exercise might be a useful tool to improve nutritional status in older adults after hospitalization. The improvements in MNA are especially relevant, taking into account that good scores in both nutritional status and physical function are considered protective factors [57,58] related to a number of adverse outcomes that could induce restricted activity [12] and social isolation of the participants [59]. These can negatively affect the autonomy and QoL of older adults and often go undetected by medical staff.

When we compared the effectiveness of both interventions, the long-term group-based branch was not any more effective than the short-term group-based branch in physical function and nutritional status. Only six weeks of group-based exercise followed by 18 weeks of home-based exercise proved to be sufficient for improving upper- and lower-limb strength, gait speed, static and dynamic balance, functional capacity, aerobic endurance, physical activity, and nutritional status. This is the first study to evaluate intervention length in this manner.

There are slight differences between the groups in their effects on QoL. Intriguingly, the LGB had a significant increase in QoL at 12 weeks, just when the group-based period finished. It should be noted that SGB had finished its group-based program six weeks before. In contrast, during the home-based period of the LGB, QoL tended to decrease. These results suggest that group-based interventions could be superior to home-based exercises in improving QoL, as proposed in patients with cancer [60] and in elderly people with sarcopenia [28]. The presence of a social element in group-based interventions could explain this difference. However, the absence of group-by-time interaction in our results makes us cautious with any interpretation; more data are needed to clarify this observation.

One of the strengths of the study is that, to the best of our knowledge, this is the first to compare two different lengths of post-hospitalization group-based multicomponent exercise interventions. These findings are relevant to design specific interventions in a hospital setting. In addition, the compliance with the interventions was good, both in the hospital and at home, which supports the feasibility of this type of program. One of the main limitations of this study is the large number (90%) of screened participants who did not accept participation in the study. This may mean that the sample is not fully representative of older people recently discharged from the hospital. The fact that many people with moderate to severe cognitive impairment or unstable cardiac conditions were excluded from the trial also limits the generalizability of the study findings. Our study may be underpowered for some variables due to the small sample size because we were unable to recruit the sample size necessary to detect significant changes in the primary outcome. However, since the effect size of time x group interaction was practically null and the significance was very close to one, it is unlikely that increasing the sample size by 28 people would cause the between-group differences to reach significance.

## 5. Conclusions

Older adults who participated in both multicomponent exercise interventions evaluated in this randomized controlled trial improved their physical function and nutritional status after hospitalization. Furthermore, we determined that six weeks of a group-based exercise intervention caused similar functional and nutritional benefits to a longer group-based intervention of twelve weeks, when both are continued at home until twenty-four weeks. These results provide valuable information for the design of practical, feasible, and cost-effective physical exercise interventions following hospitalization in older adults.

## Figures and Tables

**Table 1:** Descriptive characteristics of study participants.

	SGB	LGB
Age (years), mean ± SD	82.9 ± 5.7 (*n* = 27)	82.26 ± 5.5 (*n* = 28)
Body height (cm), mean ± SD	158.8 ± 8.4 (*n* = 27)	158.2 ± 8.5 (*n* = 28)
MMSE (0–30), mean ± SD	25.2 ± 2.3 (*n* = 27)	25.1 ± 3.3 (*n* = 28)
Barthel index, mean ± SD	86.8 ± 17.9 (*n* = 27)	84.8 ± 18.7 (*n* = 28)
Lawton and Brody index, mean ± SD	5 ± 2.5 (*n* = 27)	4.5 ± 2.3 (*n* = 28)
CCI, mean ± SD	5.6 ± 2 (*n* = 27)	5.9 ± 2 (*n* = 28)
Education level ≤ 12 years (%)	60 (*n* = 25)	60.9 (*n* = 23)
Used walking assistance device (%)	60.9 (*n* = 23)	57.7 (*n* = 26)
Entrance environmental barriers (%)	24 (*n* = 25)	19.2 (*n* = 26)
Live alone (%)	32 (*n* = 25)	37 (*n* = 27)

SGB = short-term group-based branch; LGB = long-term group-based branch; MMSE = Mini Mental State Examination; CCI = Charlson comorbidity index.

**Table 2:** Analysis of physical function and quality of life outcomes (mean ± S.D.) at 0 (pre) and 12 (post) weeks.

	SGB			LGB			η^2^
	Pre	Post	Cohen’s d	Pre	Post	Cohen’s d	
**Physical function**							
SPPB score	8.1 ± 3.4	10 ± 2.9 ***	1.269	8.4 ± 2.3	10.7 ± 1.8 ***	1.337	0.002
Hand grip non-dominant (kg)	20.8 ± 6.5	20.2 ± 7.2	0.285	21.5 ± 9.4	21.6 ± 7.8	0.035	0.050
Hand grip dominant (kg)	22.8 ± 6.8	22.5 ± 7.3	0.117	24.7 ± 9.7	25.1 ± 8.8	0.162	0.024
CST (*n* of stands)	9.3 ± 5.7	12.2 ± 6.3 **	1.446	10.4 ± 3.7	13.2 ± 4.3 ***	1.117	0.001
ACT (*n* of repetitions)	15 ± 5.9	20 ± 6.4 ***	1.756	13.4 ± 1.8	20.7 ± 1.6 ***	4.581	0.090
6mWT (m)	324 ± 135	372 ± 118 **	0.917	321 ± 117	383 ± 110 ***	1.282	0.018
8-ft TUGT (m/s)	0.27 ± 0.14	0.32 ± 0.13 **	0.870	0.29 ± 0.08	0.33 ± 0.08 **	0.807	0.003
Gait speed 8 m (m/s)	0.89 ± 0.33	1 ± 0.27 **	0.670	0.91 ± 0.19	1.03 ± 0.18 **	0.799	0.002
Fast gait speed 8 m (m/s)	1.2 ± 0.42	1.4 ± 0.38 **	0.956	1.2 ± 0.32	1.4 ± 0.3 *	0.906	0.019
Berg scale (Pts)	45.7 ± 10.3	50.5 ± 6.4 **	0.943	49.2 ± 3.3	52.5 ± 2.5 ***	1.591	0.047
**Physical activity**							
LPA (min/day)	160 ± 108	178 ± 116	0.284	132 ± 76.4	162 ± 81.6 **	0.895	0.013
MVPA (min/day)	3.7 ± 5.4	6.6 ± 9.4 *	0.501	4.9 ± 6.9	5.7 ± 7.6	0.159	0.016
Steps (nº of steps/day)	3342 ± 2849	3859 ± 3439	0.240	2722 ± 2136	3630 ± 2347 *	0.631	0.012
**Quality of life**							
EQ VAS Score	68.3 ± 15.4	77.1 ± 12.1	0.421	60.9 ± 19	72.9 ± 17.3 *	0.589	0.007
EQ-5D-5L Index Values	0.8 ± 0.22	0.88 ± 0.10	0.377	0.69 ± 0.4	0.9 ± 0.14 *	0.572	0.059

SGB = short-term group-based branch; LGB = long-term group-based branch; SPPB Score = Short Physical Performance Battery score; CST = 30-s chair-stand test; ACT = arm-curl test; 6mWT= 6-min walk test; 8-ft TUGT = 8-ft timed up-and-go test; LPA = light physical activity; MVPA = moderate-to-vigorous physical activity; EQ VAS Score = EuroQol Visual Analogue Scale score. *** *p* < 0.001, significantly different from baseline. ** *p* < 0.01, significantly different from baseline. * *p* < 0.05, significantly different from baseline.

**Table 3:** Analysis of physical function and quality of life outcomes (mean ± S.D.) at 12 (pre) and 24 (post) weeks.

	SGB			LGB			η^2^
	Pre	Post	Cohen’s d	Pre	Post	Cohen’s d	
**Physical function**							
SPPB score	10.4 ± 2.7	10.3 ± 2.9	0.099	11.6 ± 1.7	11 ± 1.9	0.809	0.001
Hand grip non-dominant (kg)	20.9 ± 7	22 ± 7	0.457	22.8 ± 8.1	23.6 ± 10.2	0.274	0.003
Hand grip dominant (kg)	23.3 ± 7	23.8 ± 7	0.313	26.3 ± 9.2	26.2 ± 10.1	0.042	0.033
CST (*n* of stands)	13 ± 6	13.13 ± 5.7	0.113	14.4 ± 4.1	14.8 ± 3.9	0.177	0.003
ACT (*n* of repetitions)	19.6 ± 6.8	21.5 ± 7.8	0.798	22.7 ± 6.5	24.9 ± 7	0.393	0.001
6mWT (m)	379 ± 125	388 ± 124	0.323	400 ± 100	405 ± 101	0.191	0.012
8-ft TUGT (m/s)	0.33 ± 0.13	0.33 ± 0.13	0.000	0.35 ± 0.08	0.34 ± 0.08	0.442	0.003
Gait speed 8 m (m/s)	1.03 ± 0.29	1.05 ± 0.27	0.163	1.06 ± 0.19	1.02 ± 0.17	0.425	0.082
Fast gait speed 8 m (m/s)	1.42 ± 0.4	1.38 ± 0.38	0.276	1.41 ± 0.31	1.48 ± 0.36	0.446	0.120
Berg scale (Pts)	51 ± 6.1	51.4 ± 7.3	0.204	52.9 ± 2	53.2 ± 2.7	0.138	0.000
**Physical activity**							
LPA (min/day)	201 ± 118	219 ± 127	0.307	177 ± 85.9	168 ± 101	0.196	0.064
MVPA (min/day)	8.1 ± 10.1	7.4 ± 7.4	0.123	6.5 ± 8.4	8.1 ± 11.7	0.311	0.057
Steps (nº of steps/day)	4541 ± 3563	4823 ± 4200	0.147	4183 ± 2365	4052 ± 2648	0.167	0.029
**Quality of life**							
EQ VAS Score	77.2 ± 12.9	75.9 ± 12.7	0.083	77.7 ± 16.5	70.8 ± 17.3	0.375	0.029
EQ-5D-5L Index Values	0.89 ± 0.1	0.89 ± 0.16	0.000	0.92 ± 0.12	0.9 ± 0.11	0.201	0.004

SGB = short-term group-based branch; LGB = long-term group-based branch; SPPB Score = Short Physical Performance Battery score; CST = 30-s chair-stand test; ACT = arm-curl test; 6mWT = 6-min walk test; 8-ft TUGT = 8-ft timed up-and-go test; LPA = light physical activity; MVPA = moderate-to-vigorous physical activity; EQ VAS Score = EuroQol Visual Analogue Scale score.

**Table 4:** Analysis of nutritional status and anthropometry outcomes at 0 (pre) and 12 (post) weeks.

	SGB			LGB			η^2^
	Pre	Post	Cohen’s d	Pre	Post	Cohen’s d	
**Nutritional status**							
MNA score	22.1 ± 2.6	25 ± 3.6 ***	1.129	21.1 ± 3.7	23.9 ± 2.5 **	0.945	0.000
MNA ranges							
Normal nutritional status *N* (%)	4 (21.1)	14 (73.7) ^a^		3 (17.6)	10 (58.8) ^b^		
Risk of malnutrition *N* (%)	14 (73.7)	4 (21.1)		11 (64.7)	7 (41.2)		
Malnutrition *N* (%)	1 (5.3)	1 (5.3)		3 (17.6)	0 (0.0)		
**Anthropometry**							
Body mass (kg)	71.2 ± 15.4	72.3 ± 15.8	0.379	71.8 ± 19.6	71.7 ± 19.9	0.046	0.017
BMI	29.1 ± 7	29.4 ± 7.3	0.235	28.8 ± 7.2	28.8 ± 7.3	0.000	0.024
Calf circumference left	35.9 ± 3.2	36.3 ± 3 *	0.456	35.3 ± 5.3	35.6 ± 5.5	0.314	0.040
Calf circumference right	36.2 ± 3	36.7 ± 3.1 *	0.526	35.5 ± 6	35.6 ± 5.6	0.088	0.012
Arm circumference	27.7 ± 5	27.5 ± 5.3	0.127	26.4 ± 5.1	26.5 ± 4.7	0.101	0.029
Waist circumference	99.5 ± 11	97.8 ± 12.9	0.374	97.3 ± 17.6	98.2 ± 17.7	0.238	0.011
Hip circumference	103 ± 13.2	103 ± 13.8	0.000	101 ± 13.6	99.4 ± 12.1	0.380	0.070
WHR	0.97 ± 0.07	0.96 ± 0.08	0.167	0.96 ± 0.11	0.98 ± 0.1	0.340	0.018

SGB = short-term group-based branch; LGB = long-term group-based branch; MNA = Mini Nutritional Assessment score; BMI = body mass index; WHR = waist-to-hip ratio. Values are means and standard deviations. *** *p* < 0.001, significantly different from baseline. ** *p* < 0.01, significantly different from baseline. * *p* < 0.05, significantly different from baseline. ^a^ *p* < 0.01; χ^2^ = 11.1. ^b^ *p* < 0.05; χ^2^ = 7.66.

**Table 5:** Analysis of nutritional status and anthropometry outcomes at 12 (pre) and 24 (post) weeks.

	SGB			LGB			η^2^
	Pre	Post	Cohen’s d	Pre	Post	Cohen’s d	
**Nutritional status**							
MNA score	26 ± 2.7	25.7 ± 1.9	0.171	24.2 ± 2.7	24.1 ± 3.8	0.037	0.001
Normal nutritional status *N* (%)	14 (87.5)	15 (93.8)		9 (62.2)	9 (62.2)		
Risk of malnutrition *N* (%)	2 (12.5)	1 (6.3)		4 (30.8)	3 (23.1)		
Malnutrition *N* (%)	0 (0.0)	0(0.0)		0 (0.0)	1 (7.7)		
**Anthropometry**							
Body mass (kg)	71.6 ± 12.3	71.3 ± 12	0.220	69.4 ± 16.4	68.8 ± 16.8	0.281	0.016
BMI	29.1 ± 5.5	29.1 ± 5.5	0.000	28 ± 5.4	27.8 ± 5.6	0.238	0.019
Calf circumference left	36.2 ± 2.8	36.1 ± 2.9	0.096	34.8 ± 4.1	35.2 ± 4.7	0.376	0.003
Calf circumference right	36.4 ± 2.5	36.3 ± 2.5	0.116	34.8 ± 4.2	34.9 ± 4.3	0.123	0.022
Arm circumference	27.1 ± 3.8	27.1 ± 3.8	0.000	25.7 ± 3.4	26.2 ± 3.7	0.514	0.009
Waist circumference	97.3 ± 11.8	97.4 ± 9	0.024	97.3 ± 15.5	98.2 ± 16	0.221	0.092
Hip circumference	102 ± 11.6	102 ± 11.3	0.000	98 ± 9.5	99.6 ± 9.8	0.502	0.008
WHR	0.95 ± 0.09	0.96 ± 0.09	0.198	0.99 ± 0.11	0.98 ± 0.1	0.272	0.067

SGB = short-term group-based branch; LGB = long-term group-based branch; MNA = Mini Nutritional Assessment score; BMI = body mass index; WHR = waist-to-hip ratio. Values are means and standard deviations.
